# Definition of a maximum threshold of direct solar radiation exposure for pedestrians of diverse walking abilities

**DOI:** 10.1007/s00484-023-02567-4

**Published:** 2023-11-04

**Authors:** Marika Tomasi, Marialena Nikolopoulou, Renganathan Giridharan, Monika Löve, Carlo Ratti

**Affiliations:** 1 Kent School of Architecture and Planning, University of Kent, Canterbury, CT2 7NR UK; 2 CRA—Carlo Ratti Associati, Turin, 10131 Italy

**Keywords:** Solar radiation exposure, Pedestrian paths, Diverse walking abilities, User profiles, Adaptation threshold

## Abstract

Since pedestrians are impacted by solar radiation differently, urban designers must evaluate solar radiation exposure of pedestrian paths adopting an inclusive approach. This paper proposes a maximum threshold of direct solar radiation exposure for pedestrians based on activity, user profile and environmental conditions, defined as the difference between the energy consumption before feeling exhausted and the energy cost of walking. Two users of diverse walking abilities, a young adult and an elderly person with mobility impairment, were characterised by metabolic activity, walking speed and maximum energy capacity. Based on the theoretical framework, the energy budget of young adults to cope with thermal stress was set as three times higher than for the elderly. This framework was used to quantify the contribution of direct solar radiation to energy balance and then classify walkability during clear-sky summer hours; the term ‘walkable’ referred to environmental conditions allowing users to walk without feeling exhausted. The methodology was tested on an open area and an urban canyon in Milan; applicability by urban designers was key in developing a simplified way to evaluate shading needs. This approach could be applied to evaluate solar radiation exposure of pedestrian paths adopting diverse user experiences as an evaluation criterion.

## Introduction

Solar radiation is a critical variable in outdoor thermal comfort because it affects the heat balance of the body (Blazejczyk et al. 1993; Hodder and Parsons 2007; Kenny et al. 2008): this is relevant when promoting outdoor activities, especially in the warm season. The 15-minute city concept argues for comfortable and safe walking environments (Abdelfattah et al. 2022); because of world population growth and urbanisation trends (UN DESA 2019), the number of people that would benefit from comfortable urban pedestrian paths is increasing*.* Santucci et al. (2020) defined walkability as the combination of health, safety, and vitality; the walking activity is strictly dependent on the quality of the public realm. In their review of definitions of walkable spaces, Forsyth (2015) cited climate as a factor influencing walking activity. Labdaoui et al. (2021) proposed a comfort walkability index as the combination of a questionnaire survey about pedestrian facilities and thermal comfort calculations. Climate walks have coupled the collection of microclimatic data at street level with surveys about the subjective perception of people, highlighting the impact of variations in dense urban morphology on thermal pleasantness (Santucci et al. 2020; Vasilikou and Nikolopoulou 2020). Literature has also presented associations of thermal stress with increasing perceived travel time (Rakha 2015) and walking speed (Bosina and Weidmann 2017; Mouada et al. 2019).

Population ageing has been considered a world demographic megatrend (UN DESA 2019). Elderly people are considered among the most vulnerable users in cities, especially during hot summer days (Dodman et al. 2022). In developing their Walkability Index for Elderly Health, Alves et al. (2020) reported that an elderly person should perform moderately intense physical activity, such as walking, for about 30 min per day. Physical activity indirectly reduces health risks due to heat stress since it improves cardiovascular capacity (Dodman et al. 2022). The main physical activity for the elderly is walking. However, mobility declines with age as well: this can be a barrier in case of uncomfortable microclimatic conditions because it would impede moving in search of restoration (Kabisch et al. 2017). Furthermore, lower walking speeds reduce the number of accessible facilities within the beforementioned 15-min radius.

Shading pedestrian paths based on their orientation and sky exposure is beneficial for pedestrians during hot clear sky conditions. Exposure to solar radiation can be addressed by modifying the urban morphology, through temporary or permanent solutions. Therefore, professionals involved in urban design and planning need to integrate the evaluation of direct solar radiation into their decision-making process.

This paper focuses on a framework to effectively evaluate the impact of direct solar radiation (DSR) on pedestrians of diverse walking abilities. It hypothesises that a maximum threshold of exposure to DSR could be related to activity, user profile and environmental conditions. Initially, the impact of incoming direct short-wave radiation on heat balance is isolated and the net thermal contribution is compared with the metabolic activity of two users, a young adult and an elderly person with mobility impairment. This proposed theoretical framework is applied to a case study in Milan, with the results presented on a DSR exposure graph, which is a simplified way to evaluate shading needs in different applications.

## Theoretical framework and methodology

The rate of DSR absorbed by the human body and its contribution to thermal stress are outlined. By comparing simulated thermal stress conditions to the definition of neutral state, the required adaptation budget is defined. Finally, this is compared to the exhaustion threshold of each pedestrian, exclusive of the energy consumed to walk at the appropriate speed.

### Direct solar radiation contribution to thermal comfort

The thermal interaction of the human body with the surrounding environment is described in the following equation (ASHRAE 2005):

1 $$M- W ={q}_{sk}+{q}_{res}+S$$

where

M: rate of metabolic heat production (W/m^2^).
W: rate of mechanical work accomplished (W/m^2^).
q_sk_: total rate of heat loss from skin (W/m^2^).
q_res_: total rate of heat loss through respiration (W/m^2^).
S: total rate of heat storage (W/m^2^).

On the left side of Eq. (1), M is the total metabolic rate within the body (sum of M_act_ and M_Shiv_ for activity and shivering respectively) while W is the energy that might be expended as external work. Since this paper is focused on warm/hot microclimatic conditions, M_shiv_ will be considered null. The external work will also be ignored because the ratio W/M is usually less than 0.10, and null in case of walking on a flat surface (ASHRAE 2005; Jendritzky 1990). The right side of the equation describes how the net heat production (M-W) is transferred to the environment through the skin surface and respiration. S is any surplus or deficit of energy stored, which causes the body’s temperature rising or decreasing; nevertheless, it will be ignored because of the limited thermal storage capacity of the body (Alahmer et al. 2012). Therefore, for the purpose of this research, the metabolic rate derived from muscular activity is defined as the sum of the heat transferred through the skin and respiratory tract via convection, radiation, and evaporation.

Exposure to DSR impacts the energy balance of a pedestrian; its contribution can be isolated and will be hereinafter referred to as R^*^. When exposed to DSR, a body absorbs a fraction of it, which is equal to:

2 $${R}^{*}=I\cdot {a}_{k}\cdot {f}_{p}$$

where

I: DSR on a surface perpendicular to the sun’s rays (W/m^2^).
a_k_: absorption coefficient of the irradiated body surface area for short-wave radiation.
f_p_: surface projection factor.

The standard value adopted for a_k_ is 0.7 (ASHRAE 2005). The surface projection factor refers to the area effectively exposed to solar radiation^1^ and can be calculated as a function of solar altitude (γ) as reported by Jendritzky (1990):

3 $${f}_{p}=0.308 \cdot \mathrm{cos}\left(\gamma \left(0.998- {\gamma }^{2}/50000\right)\right)$$

In warm conditions, mean radiant temperature (T_mrt_) is the parameter with the largest effect on human thermal comfort (Matzarakis et al. 2010). It corresponds to ‘the uniform temperature of an imaginary enclosure in which radiant heat transfer from the human body equals the radiant heat transfer in the actual nonuniform enclosure’ (ASHRAE 2005). Both long- and short-wave radiations are included in its calculation. The critical impact of DSR on T_mrt_ during daytime has been reported by previous research, in particular in urban canyons (D E V S et al. 2019). Additionally, short-wave radiation largely varies under clear skies, and in summer, is normally higher (Ji et al. 2022). Because of the moving position of the sun, and the blocking effect of urban morphology, short-wave radiation collected by surfaces can substantially change. Consequently, changes in T_mrt_ can be recorded within a few meters radius; for this reason, Naboni et al. (2017) defined it as a ‘spatial metric’, which makes it relevant for urban design. Jendritzky (1990) isolated the contribution of DSR on T_mrt_:

4 $${T}_{mrt}^{*}={\left[{T}_{mrt}^{4}+\frac{{f}_{p}\cdot {a}_{k}\cdot {I}^{*}}{\left({\varepsilon }_{p}\cdot \sigma \right)}\right]}^{0.25}$$

where

T^*^_mrt_: mean radiant temperature in case of DSR exposure.^2^
T_mrt_: mean radiant temperature with no DSR exposure.^3^
ε_p_: emission coefficient (standard value = 0.95 (ASHRAE 2005)).
σ: Stefan-Boltzmann constant (5.67 ∙10^–8^ W/(m^2^∙K^4^)).

Numerous models have been proposed to describe thermal comfort as a function of meteorological variables. The Universal Thermal Climate Index (UTCI) was developed specifically to describe the thermal state of a human body in a non-steady state, which suits the description of a pedestrian walking outdoors (COST Action 730 n.d.; Nikolopoulou 2011). Equation (5) defines UTCI as the sum of air temperature and an additional contribution that depends on four meteorological variables (Bröde et al. 2012):

5 $$UTCI\left({T}_{a},{T}_{mrt},{v}_{a},{p}_{a}\right)= {T}_{a}+offset({T}_{a},{T}_{mrt},{v}_{a},{p}_{a})$$

where

T_a_: air temperature.
v_a_: wind speed.
p_a_: humidity (expressed as water vapour pressure).
T_mrt_: mean radiant temperature.

Bröde et al. (2012) proposed a regression function to calculate UTCI through the difference between T_a_ and T_mrt_ (for given v_a_ and p_a_ values, i.e., ‘reference conditions’); they found that a 10 K increment in T_mrt_ corresponded to a 3 K increment in UTCI, which highlights the influential contribution of DSR on outdoor thermal stress.

### Building the DSR exposure graph

This section presents a model to isolate the contribution of DSR to thermal stress. This contribution is shaped as a DSR exposure graph delivered to urban designers to evaluate the user experience provided by outdoor spaces and exposure to DSR, with particular attention to pedestrian paths. The core assumption is that in absence of thermal stress, the metabolic heat produced during the performed activity is balanced by the heat exchanged with the surrounding environment. Once the surrounding environment presents uncomfortable conditions, the body attempts to reinstate the neutral conditions (no thermal stress) by releasing the additional heat, mainly via evaporation and convection.

**Fig. 1:**
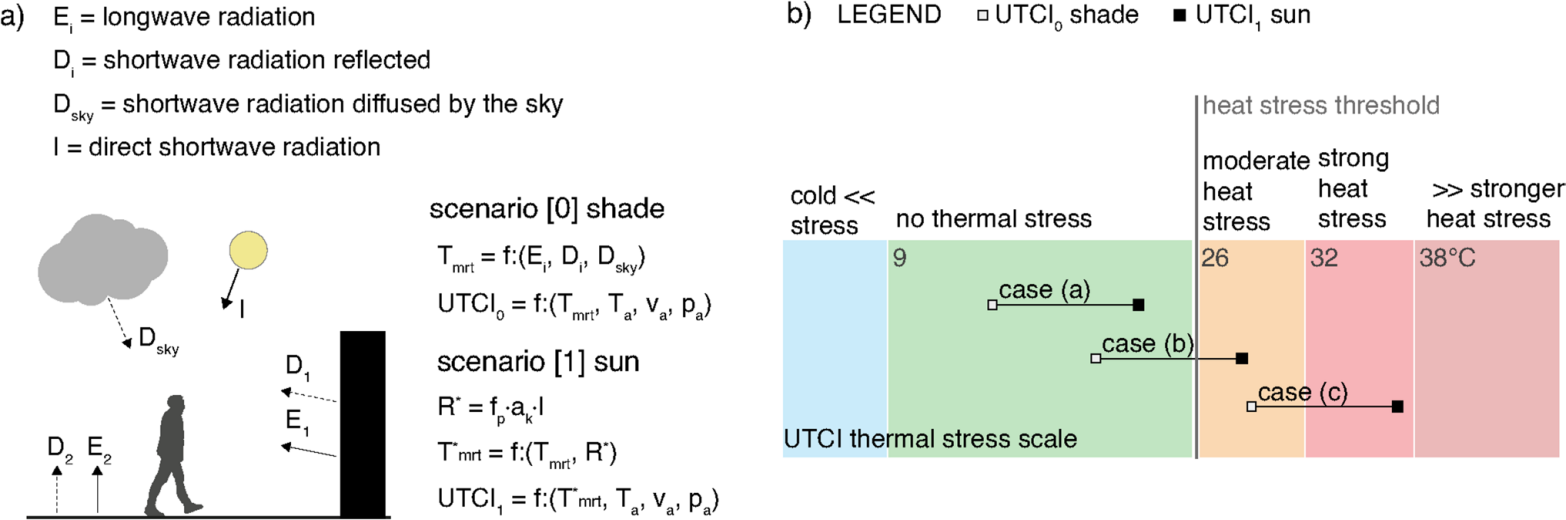
(**a**) Radiation fluxes considered to calculate T_mrt_ and UTCI in shade, scenario [0], and under the sun, scenario [1] (adapted from Naboni et al. 2019); (**b**) Position on the UTCI scale of the three defined cases before and after DSR exposure: in case (**a**), the UTCI value does not cross the heat stress threshold after the DSR exposure, therefore the person is not in heat stress even under the sun; in case (**b**), the UTCI value crosses the heat stress threshold; in case (**c**), the person is already in heat stress even in shaded conditions

To evaluate the impact of DSR exposure on pedestrians, two scenarios were simulated, users in full shade and under the sun. At first, UTCI was calculated for a pedestrian in shaded conditions, defined as scenario [0]; then, the term R^*^ in Eq. (2) was calculated for isolating the DSR contribution to heat balance, as proposed in the previous section. The contribution of DSR to T_mrt_ was determined via Eq. (4), and UTCI was simulated (Fig. 1a); these values referred to scenario [1], in which the user was directly exposed to the sun. UTCI values for scenarios [0] and [1], indicated as UTCI_0_ and UTCI_1_, were subsequently assigned to three different cases, as graphically illustrated in Fig. 1b:

- case (a): the user is in no thermal stress conditions in both scenarios;
- case (b): the user is entering in heat stress conditions after being exposed to direct solar radiation;
- case (c): the user is in heat stress conditions already in the fully shaded scenario.

Based on the presented model, the increase in thermal stress value (ΔUTCI) is due to R^*^. In order to evaluate the effect of DSR exposure on pedestrians, at first the shaded conditions must be assessed in terms of thermal stress. For each set of microclimatic conditions (T_a_, v_a_, p_a_, T_mrt_), the amount of absorbed heat necessary to reach the heat stress threshold (UTCI = 26 °C) when in shade was calculated; this speculative variable was defined as R_0_. To calculate R_0_, a linear relationship was assumed between R^*^ and ΔUTCI. Although this relationship is not perfectly linear, the linear regression analysis performed on the available dataset validated this approximation for values close to the heat stress threshold (Appendix A). Therefore, for each hour, UTCI_0_ was associated with the correspondent R_0_ value using Eq. (6):

6 $${R}_{0}=\frac{{(UTCI}_{0}-26) \cdot {R}^{*}}{\Delta UTCI}$$

where

R_0_: equivalent absorbed solar radiation in shaded conditions.
UTCI_0_: thermal stress value in shaded conditions.
∆UTCI = (UTCI_1_ – UTCI_0_): increase in thermal stress value due to solar radiation exposure.

**Fig. 2:**
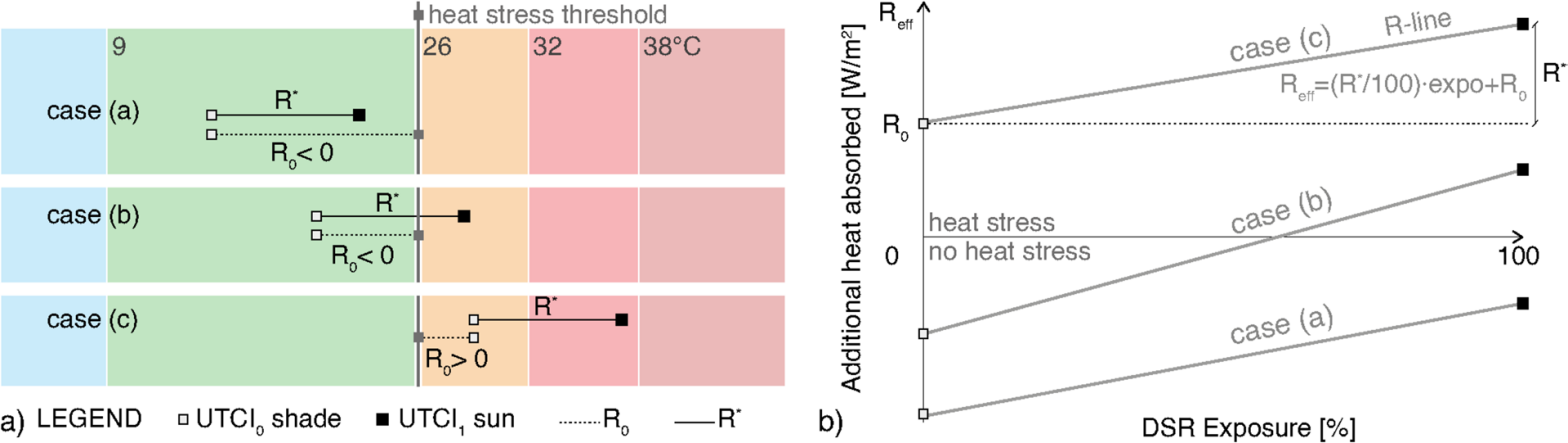
(**a**) Graphical definition of R_0_: at given microclimatic conditions, it is the equivalent absorbed DSR that represents the gap between the UTCI value in shaded conditions and the heat stress threshold (UTCI = 26 °C); (**b**) DSR graph weighting R^*^ based on the percentage of DSR exposure and adding R_0_ to consider the effective heat load on the human body in fully shaded conditions

In this way, for each hour, an equivalent heat load was associated with the gap between UTCI_0_ and the threshold value indicating thermal stress, i.e., 26 °C, in both directions. The outcome of Eq. (6) was either a positive or a negative value (Fig. 2a). In cases (a) and (b), R_0_ was the equivalent amount of energy needed to exit the no thermal stress zone; it was therefore a negative number corresponding to the energy budget that could be absorbed by the body while remaining in comfortable conditions. On the contrary, in case (c), R_0_ was a positive number and represented an ‘equivalent’ solar radiation energy as if the pedestrian was accumulating heat even though not being exposed to DSR. In this way, the heat stress condition was represented in the graph. Effective solar radiation (R_eff_) was consequently defined as the amount of energy that would affect the human body at given conditions, moving it away from the no thermal stress zone, and corresponded to:

7 $${R}_{eff}={{R}^{*}+R}_{0}$$

During a trip in the urban environment, it is rare to walk only under the sun or in shade; instead, it is most common being exposed to DSR for a certain time. To consider the impact of the sun in relation to various percentages of DSR exposure, for each set of microclimatic conditions, an R-line was defined using Eq. (8). The contribution of R^*^ was weighted based on the percentage of exposure to DSR: 0% corresponded to a trip performed in total shade and 100% totally under the sun.

8 $${R}_{eff}= \frac{{R}^{*}}{100}expo+{R}_{0}$$

where expo: percentage of exposure to DSR (from 0 to 100%).

A DSR exposure graph was built as a collection of R-lines: it illustrates the effective impact of DSR added to the heat energy balance of a person as a result of an increase in T_mrt_, considering the remaining meteorological variables included in the UTCI calculation (T_a_, v_a_, p_a_) as constant. Figure 2b provides the graphical representation of the DSR exposure graph.

### Profiling users of diverse walking abilities

This section aims to delineate the differences in metabolic heat production among diverse pedestrians. Two user profiles are defined, a young adult and an elderly person with mobility impairment.

To maintain body temperature within a normal range, the heat produced by metabolic activities must be dissipated, transferring it to the surrounding environment. This mechanism mostly happens at the skin level, so it is often convenient to express the metabolic activity in heat production per unit area of skin (ASHRAE 2005). The body surface area was defined as function of body mass and height of the individual by DuBois and DuBois in 1916 (ASHRAE 2005). A resting person produces about 100 W; the DuBois area of an average European man is 1.8 m^2^ (height = 1.73 m, body mass = 70 kg). Based on these specifics, the standard metabolic rate of a resting person, sitting quietly, is defined as 58.1 W/m^2^ and is called 1 met. Elderly people have a lower resting metabolic rate, which based on previous research was set to 0.75 met (Hall et al. 2013, 2014).

#### Energy consumption in walking

Focusing on pedestrians’ activity, the typical walking speed for each user group was defined. Then, each walking speed was associated with the appropriate energy cost for the corresponding user profile, assessed from literature. The term ‘energy cost’ describes the physiological work in performing one activity; it is derived from the rate of oxygen consumption and, once adjusted based on activity and individuals, it allows comparative analysis (Van Swearingen and Studenski 2014).

Based on the review by Bosina and Weidmann (2017), a walking speed of 1.34 m/s was adopted for young adult pedestrians. The corresponding metabolic heat production was obtained by values proposed by ASHRAE (2005) through linear interpolation, 2.9 met. Oxley et al. (2004) reported walking speeds of pedestrians with different assisting devices and levels of physical ability. The average walking speed was 0.79 m/s, which is consistent with literature about the effect of age on pedestrian speed (Pinna and Murrau 2018). Van Swearingen and Studenski (2014) presented three curves of the energy cost of walking based on the walking speed of older adults with impaired motor skills; a value of 0.225 ml O_2_/kg/m was extracted from the curve describing the moderately slow pedestrians, as it was associated with the walking speed of 0.79 m/s. After converting the selected value in the appropriate unit,^4^ the equivalent metabolic heat production by an elderly user for walking was set at 3 met, as 1 met is equal to 3.5 ml O_2_/kg/minute (Jetté et al. 1990). Even though consistent with previous studies (Hall et al. 2013), this value is higher than the results obtained by Martin et al. (1992), which reported values around 2.7 met for comparable walking speeds; this may be due to the different physical conditions of participants since the latter study selected older pedestrians with no physical impairment.

Table 1 summarises the values presented above. The energy cost of walking for young adults and elderly pedestrians is similar: that is because it is calculated for the appropriate walking speed of each group, while the energy cost for the elderly is higher than younger pedestrians when calculated for the same walking speed (Martin et al. 1992).

**Table 1:** Energy cost of resting and walking for two selected users

User group	Energy cost of resting [met]	Walking speed [m/s]	Gross energy cost of walking [met]
Young adults	1.00^1^	1.34^2^	2.9^1^
Elderly	0.75^3^	0.79^4^	3.0^5^

1. ASHRAE (2005), 2. Bosina and Weidmann (2017), 3. Hall et al. (2013, 2014), 4. Oxley et al. (2004), 5. Van Swearingen and Studenski (2014)

#### Maximum energy capacity threshold

As discussed in before, the underlying assumption is that the additional thermal energy R_eff_ is dissipated by the body through skin and respiration. To define a threshold that would indicate walking as an exhausting activity for pedestrians, the value R_eff_ was compared to the physical effort that would induce the body to dissipate the same amount of additional heat. This comparison allowed us to introduce the concept of maximum energy capacity in our methodology.

**Fig. 3:**
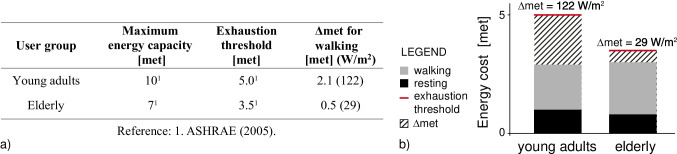
(**a**) Definition of walking adaptation thresholds for two selected users as the difference between the exhaustion metabolic threshold and energy cost of walking; (**b**) Energy cost of activities for two user profiles with relative energy budget available to cope with additional heat load (‘walking adaptation threshold’)

ASHRAE (2005) reports that the maximal capacity to use oxygen (‘maximum energy capacity’) depends on various factors such as gender, age, physical condition. For this research, the adopted values of maximum energy capacity were 10 met for young adults and 7 met for the elderly. These values refer to a 35-year-old who does not exercise and a 70-year-old man, respectively (ASHRAE 2005, pp 8.6–7). A person can continuously perform physical activity as long as 50% of their maximum energy capacity is not reached (ASHRAE 2005). Therefore, the difference between 50% of maximum energy capacity, hereinafter defined ‘exhaustion threshold’, and the walking energy cost for each pedestrian (refer Table 1) was defined as ∆met (Fig. 3). Within the proposed framework, ∆met was defined as the energy budget (‘adaptation threshold’) available for the user to cope with outdoor thermal stress before feeling exhausted because of the environmental conditions. Therefore, ∆met for walking was set to 2.1 and 0.5 met for young adults and the elderly respectively.^5^ However, if young adults and the elderly were at rest, the adaptation thresholds were 4.0 and 2.8 met respectively.

**Fig. 4:**
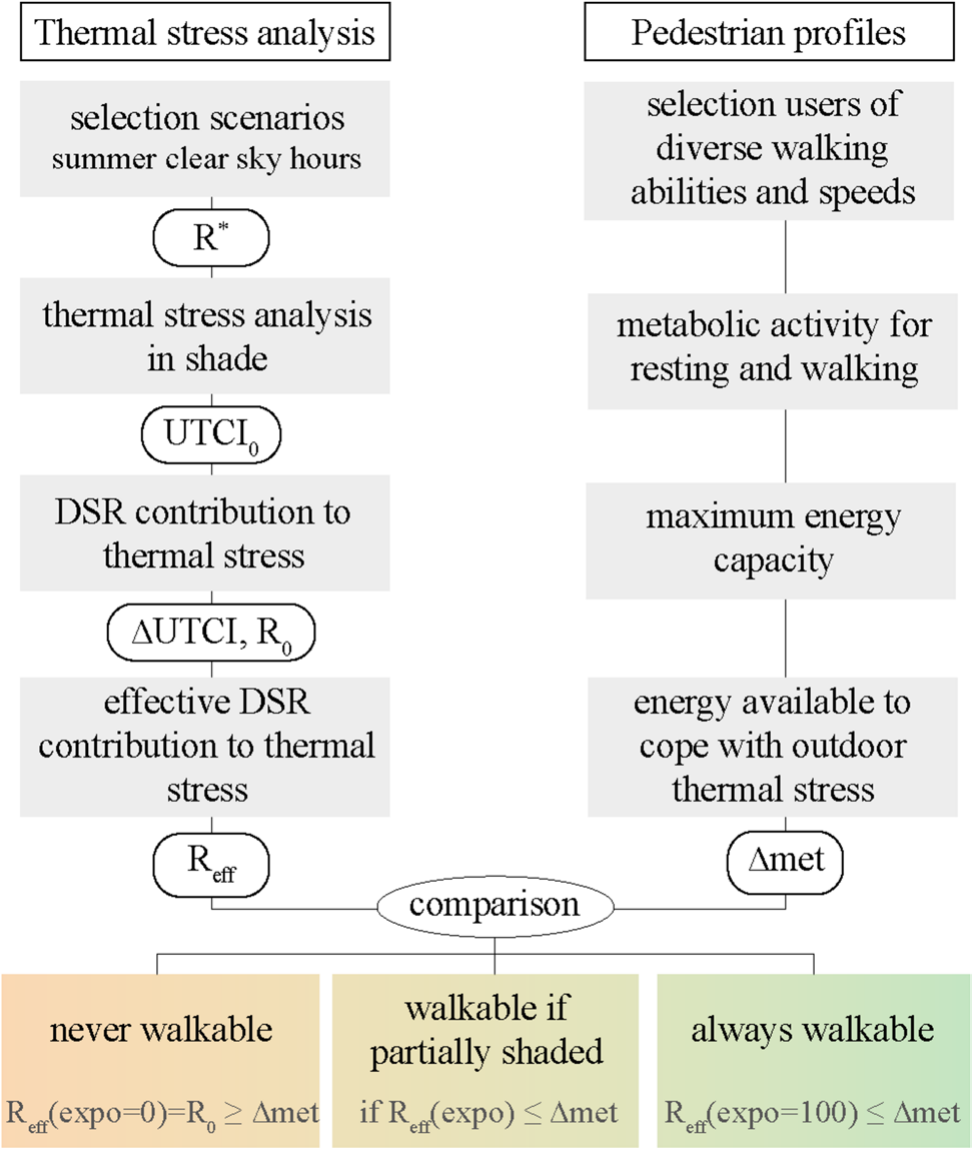
Methodological scheme illustrating the comparison between DSR and metabolic activity of pedestrian profiles. The outcome of each step is presented below the relevant description; at the end, the classification of analysed hours in three categories is presented. The term ‘walkable’ refers to a thermal condition in which the DSR exposure would not make the considered pedestrian feel exhausted

Walking in the urban environment, users regularly cross different DSR exposure levels. The weighted average metabolic rate was thus introduced, in line with the analogy of metabolic activity and additional thermal load. According to ASHRAE, if activities are alternated frequently over time, the weighted average metabolic rate is ‘generally satisfactory’ (2005, p. 8.7). For application purposes, the investigation of each hypothetical trip during the selected hour was therefore weighted based on how much time a pedestrian would be exposed to or screened from DSR. This simplified model was created to evaluate thermal conditions in the shade and under the sun at a specific moment in time. Therefore, the weighting operation did not consider transitioning from shade to sun (and vice versa), the two conditions were assumed as an instantaneous variation in the heat exchange with the surrounding environment. For the purpose of this research, the term ‘walkable’ was used to define thermal conditions allowing the considered pedestrian to walk under a percentage of DSR exposure without feeling exhausted. Using the DSR exposure graph, the R_eff_ value corresponding to the percentage of time spent under the sun was compared with ∆met for the selected user, and three outcomes were possible. At the given microclimatic conditions, based on the DSR exposure and metabolism of the pedestrian, a trip could be categorised as always walkable (i.e., always below the exhaustion threshold) as long as R_eff_ was lower than ∆met even under the sun; never walkable, in case ∆met was reached already in the shade (meaning exhausting heat load even if totally screened from DSR); and walkable if partially shaded, where the resulting percentage of DSR exposure corresponded to the value on x-axis (expo) where the R-line (Fig. 2b) intercepted ∆met. Figure 4 summarises the presented methodology and possible categorisation outcomes.

## Applying the methodology to a case study

The methodology was tested using an open area in the city of Milan (Italy) as a case study. Being in the Po valley, its climate is classified as Cfa—humid subtropical climate, with hot and humid summers. Input weather data were downloaded from the Energy Plus website (EnergyPlus n.d.). The selected station was Milano-Linate 160800 (IGDG) – location: N 45° 25', E 9° 16'. At the end of year 2021, out of the about 1.4 million residents, 12.9% were over 75 years old (Comune di Milano 2022), which highlights the necessity to delineate diverse user profiles in urban planning practices for this city.

Calculations were performed on an hourly basis because meteorological variables in the dataset were reported every hour, and pedestrians were assumed to walk short distances within the urban environment. DSR exposure in Milan was analysed for hours considered suitable to simulate the contribution of DSR to the human energy balance; the selection process is here reported. All summer days were considered, from June 21^st^ to September 22^nd^. This selection included the summer solstice day, as well as the hottest and typical weeks based on.epw statistics (6–12/7 and 13–19/7, respectively). The hours selected range from 8 am to 5 pm, namely 9 am to 6 pm DST (daylight saving time, UTC + 2); hours that registered null DSR were removed. Only clear sky days were considered: the dataset available reported total sky cover in tenths and values from 0 to 2 were selected, as correspondent to the equivalent lowest category of cloudiness in oktas (World Meteorological Organisation n.d.). In the end, 274 h were analysed; all combinations hour of the day/month were covered.

As a proof of concept, the methodology was tested on an open area. This allowed assuming surface temperature as equal to air temperature, avoiding additional complexity from the surrounding surfaces. Simulations were performed using Ladybug tools (v1.5.0) in Grasshopper, the visual scripting interface for Rhino software (Grasshopper n.d.; Ladybug n.d.; Rhinoceros n.d.). R^*^ was calculated for all selected hours using Eq. (2). DSR was obtained from the variable ‘direct normal radiation’ in.epw file, which corresponds to the amount of solar radiation received from a surface perpendicular to the sun’s rays in the 60 min before the associated time (U.S. Department of Energy 2002).^6^ Solar altitudes were derived from the *Sunpath* component and were used to calculate f_p_. T_mrt_ in shaded scenarios was calculated using the *OutdoorSolarMRT* component, by setting to zero the body fraction exposed to direct sunlight. This component uses the SolarCal model to calculate T_mrt_ considering shortwave solar radiation and estimating longwave radiant exchange with the sky. Then, the additional contribution of DSR to T_mrt_ was calculated using Eq. (4). It should be noted that f_p_ is formulated to be independent of the user’s orientation to the sun; therefore, results obtained with this method could be used for pedestrians walking in any direction. The two T_mrt_ values were inserted into the *UTCI* component, together with the other meteorological variables required, derived from the.epw file. For each calculated hour, the resulting ΔUTCI was compared against R^*^; then, R_0_ was calculated to determine the equivalent solar radiation value in fully shaded conditions and the DSR exposure graph was compiled for young adults and elderly. Finally, applications of results were presented.

## Results

### Solar radiation absorbed by pedestrians

**Fig. 5:**
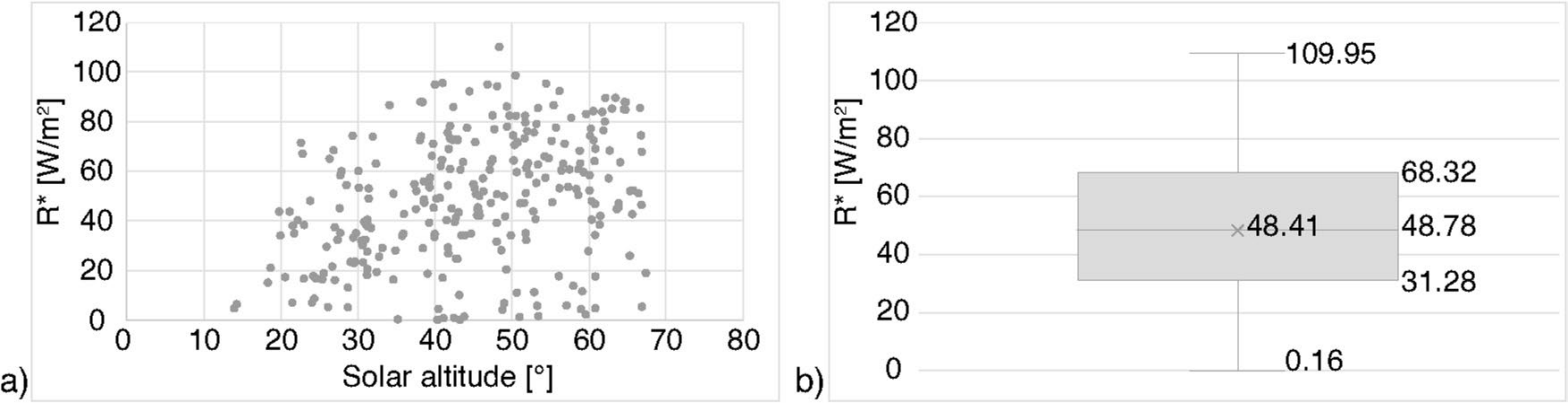
(**a**) DSR absorbed by the body (R^*^) compared against solar altitudes; (**b**) Box plot showing the distribution of DSR absorbed by the body (R.^*^)

Figure 5 reports relevant statistics about the outcome of the calculation of R^*^. As expected, no increasing trend in the DSR absorbed by a pedestrian at higher solar altitudes could be observed (Fig. 5a). This is due to opposite trends of I and f_p_, both included in the calculation of R^*^: while high values of I are generally recorded in the central part of the day (when γ is high), f_p_ decreases with higher solar altitudes. Therefore, high variability in R^*^ was found. The average value of R^*^ was 48.4 W/m^2^ and increased to 51.4 W/m^2^ if values < 58.1 W/m^2^ (0.1 met) were not considered (17 in total, 6% of analysed hours). The maximum value of R^*^ was 109.95 W/m^2^ and corresponded to 2^nd^ September at 12 pm DST.

#### Thermal comfort analysis via Ladybug

T_mrt_ was calculated in shaded conditions and under the sun, and the corresponding UTCI values were obtained. Since wind speed was reported as null for 126 h, the minimum value allowed by the UTCI formulation, which is 0.5 m/s, was used in those cases. In total, 44 h always resulted in the UTCI no thermal stress zone, even if completely exposed to solar radiation (case a). Among these, in two cases, UTCI was less than 9 °C under the sun (i.e. slight cold threshold): they corresponded to 3–4 pm DST of August 21^st^, which reports direct normal radiation values < 10 Wh/m^2^, T_a_ < 16 °C and high wind speed (9.6 to 10.9 m/s). Conversely, 210 h resulted in heat stress already in shaded conditions, at various levels: 135 moderate, 73 strong, and 2 very stronger heat stress (case c).

### DSR exposure graph for an open area in Milan

**Fig. 6:**
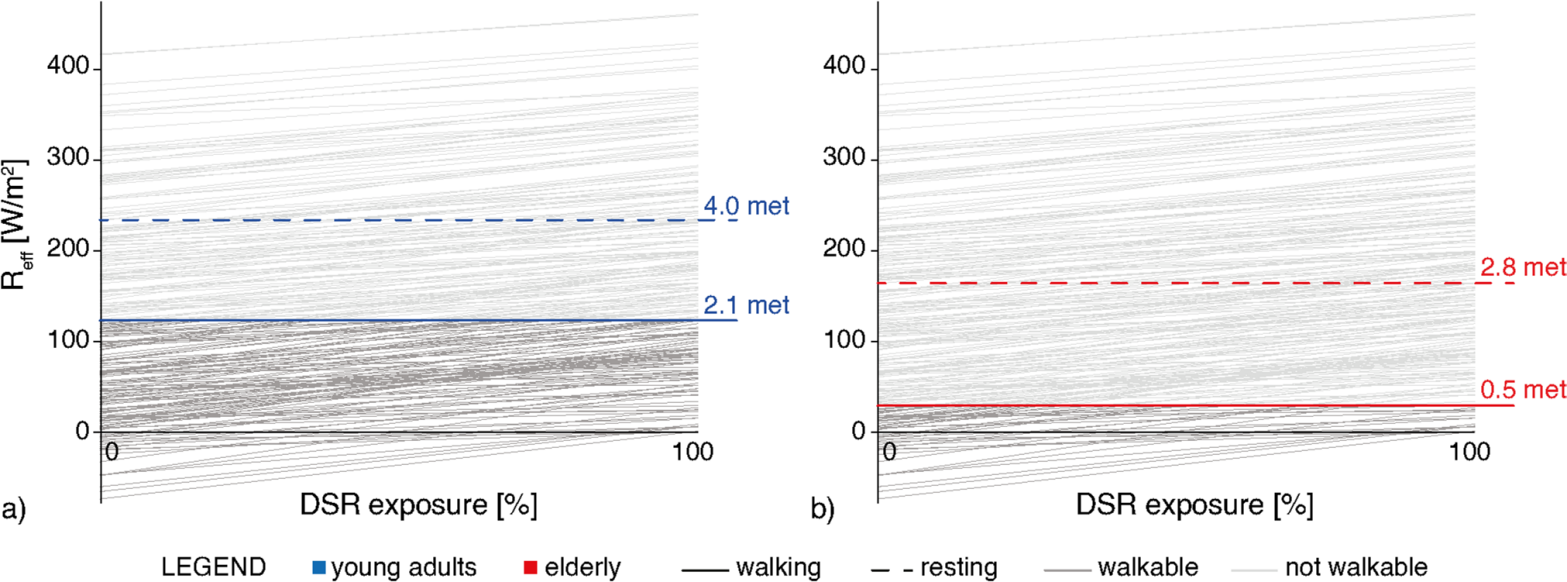
Impact of DSR exposure on young adults (**a**) and elderly (**b**) in an open area in Milan; ∆met is represented as solid and dotted lines for pedestrians walking and resting respectively. Each R-line represents one analysed hour; hours below the walking adaptation thresholds are represented as dark lines

Results of the linear regression analysis between ΔUTCI and R^*^ were in good agreement with the hypothesis of approximating the impact of DSR on outdoor thermal stress via linear equation, as reported in Appendix A. Therefore, R_0_ and R_eff_ were calculated with Eqs. (6) and (7), respectively. Figure 6 reports the resulting DSR exposure graph (references for its interpretation are presented in Fig. 2). Every R-line represents one analysed hour; R-lines corresponding to hours always below the heat stress threshold were removed from the graph. For every hour that resulted R_eff_ below the adaptation threshold while walking was represented with a dark line; if it intercepted the walking adaptation threshold, it was coloured only for the length below the relevant limit.

**Fig. 7:**
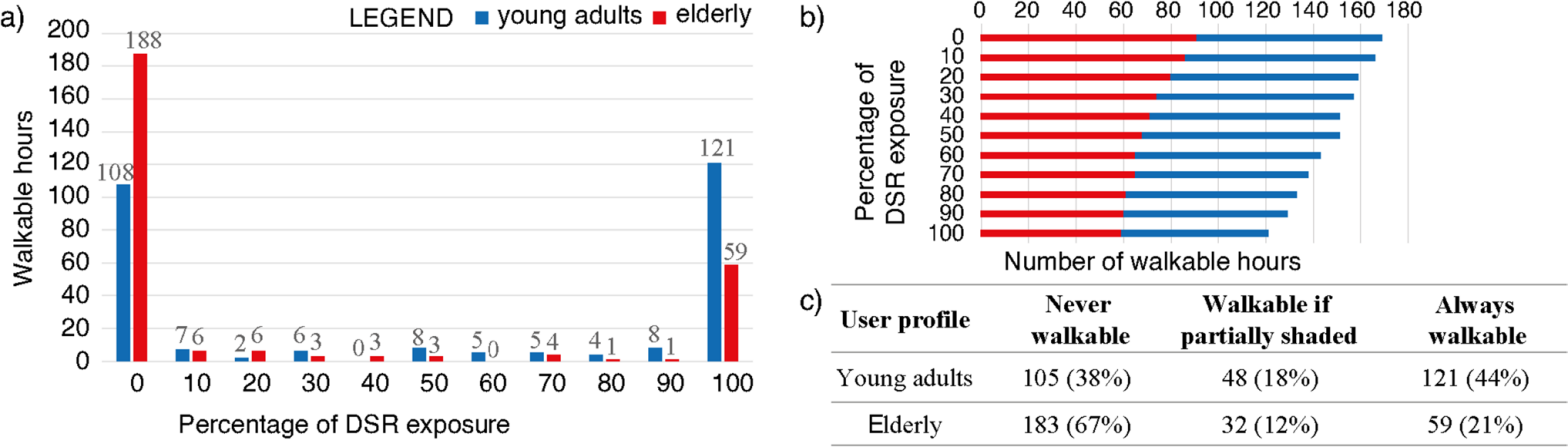
Synthesis of results of the DSR exposure graph reported in Fig. 6; (**a**) subdivision of analysed hours in bins (bin N refers to N ≤ expo < N + 1); (**b**) staked bars to highlight trend in walkability to the change of DSR exposure; (**c**) summary of results based on the three proposed categories

Results obtained from the DSR exposure graph are synthesised in Fig. 7. Hours were allocated into 10% bins representing the recommended DSR exposure percentage for each user profile in that specific combination of microclimatic and environmental conditions. The bin named ‘50’ collected expo values in the range of 50 ≤ expo < 60%. The aggregated evaluation of 274 sets of microclimatic conditions led to drawing some conclusions about environmental conditions through the summer season. For young adult pedestrians, open areas in Milan resulted in being always walkable for 121 h. On the other hand, 105 h were too much challenging for their metabolic system, also in shaded conditions. This is a critical result because based on our calculations, walking outdoors would not be recommended for 38% of the analysed hours under clear skies in summer. The remaining 48 h were distributed almost homogeneously among different percentages of DSR exposure. For elderly pedestrians, the situation was more critical, as expected: walking under the sun would have been possible only for 59 h (21%), and additional 32 h would require different percentages of shade.

**Fig. 8:**
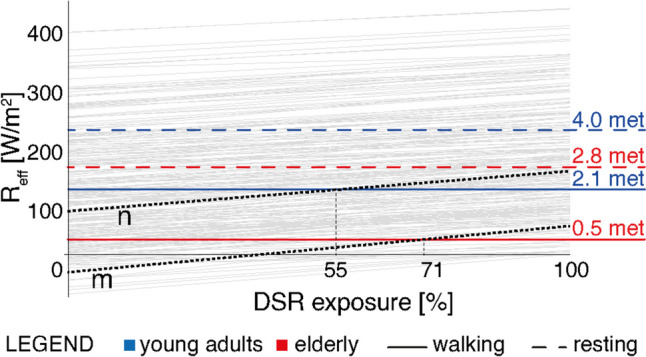
Two case study hours are isolated to illustrate the effect of DSR exposure on the two users in selected conditions

In addition to statistics presented in Fig. 7, the DSR exposure graph can be used to evaluate specific user experiences: two cases are highlighted on the same graph and reported in Fig. 8. Additionally, the step-by-step calculation process for both cases is reported in Appendix B. Line ‘m’ refers to 10 am DST on July 6^th^. UTCI_0_ value (in the shade) results as 24.6 °C; with ΔUTCI = 3.8 K, in sunny conditions, the user comes out of the ‘no thermal stress zone’ and enters the ‘moderate heat stress’ state. Based on R_eff_ values, if exposed to DSR in the analysed microclimatic conditions, elderly pedestrians can walk for up to 71% of the time under the sun without feeling exhausted. Therefore, if a trip of ten minutes is hypothesised, this means that they would need at least three minutes in the shade; instead, young adults could complete the trip without the need to find shade. Line ‘n’ refers to a more extreme case. Microclimatic conditions refer to August 27^th^ at 12 pm DST, and UTCI values range from 29.1 to 31.9 °C. In these conditions, the young adult pedestrian also needs to have a break after walking in the sun; 55% is the maximum amount of time to walk exposed to solar radiation without feeling exhausted. Elderly pedestrians would not be recommended to walk outdoors in these conditions, since the adaptation threshold is already exceeded in the totally shaded scenario.

### Application to urban design practice

**Fig. 9:**
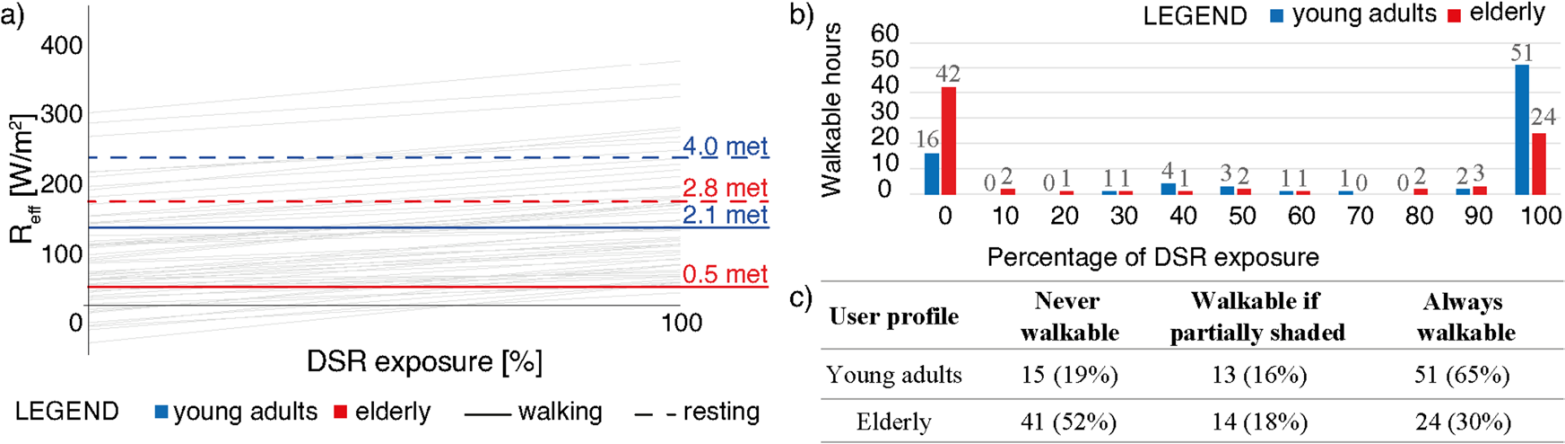
DSR exposure impact on young adults and elderly in an urban canyon of H/W = 1.0 in Milan. (**a**) DSR exposure graph, where ∆met is represented as solid and dotted lines for pedestrians walking and resting respectively. Each R-line represents one analysed hour; (**b**) subdivision of analysed hours in bins (bin N refers to N ≤ expo < N + 1); (**c**) summary of results based on the three proposed categories

The proposed methodology was developed considering an open area to avoid the additional complexity from modelling surrounding surfaces. The same procedure could be applied in an urban setting by modifying urban morphology descriptors. In this section, an application of the DSR exposure graph in urban design practice is presented. Different aspect ratios (H/W) affect outdoor thermal comfort, resulting in different sky view factors (SVF) for people walking into the urban canyon (Nouri et al. 2017). An urban canyon 15 m wide and of H/W = 1.0 was modelled; a pedestrian was positioned one meter from the closest building. The SVF from that position was calculated through the *HumanToSky* component in Ladybug and resulted in 0.3 (*sky exposure factor* in Ladybug tools). Ground reflectance was set to 0.12 to simulate paved sidewalks. The surface temperature was calculated via Rayman (Matzarakis et al. 2010) and imported in Ladybug. To simulate the design goal of investigating walkability during a specific time of the day, morning hours were selected, specifically from 9 to 11 am. A total of 79 h were analysed, and the results are presented in Fig. 9.

Analysing morning hours is important for walkability in cities since microclimatic conditions are generally more comfortable than the rest of the day. Results obtained through the proposed methodology show that 30% of the hours are categorised as always walkable for elderly pedestrians. A valuable result for urban designers is that, if 70% of pedestrian paths would be shaded during morning hours, walkability for elderly pedestrians would increase by 42%, reaching 43% of walkable morning hours from the dataset (34 h). This emphasises how paths not completely shaded would have anyway an impact on improving walkability.

## Discussion

This paper has presented a methodology to evaluate the impact of DSR exposure on pedestrians of diverse walking abilities on hot clear summer days. A key finding was the focus on thermal stress and physical effort, because recommended DSR exposure times had been proposed based on health risks due to overexposure to ultraviolet (UV) radiation, such as skin damage (Diffey 2018; WHO 2003). The application of this methodology to urban design workflows makes it valuable to evaluate user experiences in design proposals, and accordingly, address emerging issues. The DSR exposure graph could inform urban designers of relevant percentages of shade to achieve in design proposals; allow a multi-perspective analysis of a specific urban morphology (e.g., an urban canyon) by changing input settings; and compare user experiences in different locations evaluating thermal stress through DSR exposure. The application of DSR exposure thresholds obtained through the presented workflow is not limited to urban design practice. Expanding the scale of analysis, they can be included in urban planning requirements, for both existing and new development areas. Comfortable public outdoor spaces must be equally distributed in the city because of their key role in citizens’ well-being: public areas are a refuge during extremely hot days, especially for lower-income residents, that cannot afford A/C systems (Aljawabra and Nikolopoulou 2018). Other applications include public communication at the city level of recommended maximum exposure time to DSR; the definition of minimum distances between relief spots, to allow vulnerable users to have a break decreasing the average energy cost of a trip; measuring the effectiveness of design proposals from the users’ perspective. The proposed workflow aims to support urban designers in making decisions, yet they must select the final threshold to be considered in their design, as explained by Hendel et al. (2020).

Ladybug tools were selected because the computational time required for microclimatic analysis is shorter compared to other simulation tools, a key consideration in architectural practice. The downside of fast calculation lies in the assumptions made at the beginning of the process. Especially when the workflow was applied to an urban canyon (Fig. 9), T_mrt_ in shaded conditions did not take into consideration long- and reflected short-wave radiation emitted by surrounding vertical surfaces, which could have an effect on T_mrt_ (Salvati et al. 2022). Furthermore, the surface temperature was calculated through a different software (Rayman). The potential usability by urban designers and the possibility to perform extensive analysis with acceptable computational time overruled the need for more accurate results. It is worth highlighting that the methodology is independent of the tool used to simulate microclimatic variables; if more detailed analysis was required, software with higher accuracy in results could be used, focusing on a limited number of simulation scenarios, to avoid computational times increasing excessively.

Drawing attention to more inclusive cities is a major objective of this paper. As reviewed in the theoretical framework, standards and comfort scales have been developed considering the physiology of male adults. Nevertheless, cities are required to adapt to a diversified society, especially considering vulnerable people. In this paper, young adults and elderly pedestrians with impaired mobility were analysed, however, considering further user groups is essential. Females, on average, have 30% less maximum energy capacity than men (ASHRAE 2005), but with a lower metabolism (Ferraro et al. 1992), Δmet would not be expected to vary substantially, although further studies will be required. An additional user group that could be considered in the future is children and toddlers, who have higher resting metabolic rates than adults but lower walking speeds (DeJaeger et al. 2001).

A limitation of this work is that the UTCI scale was not adapted to metabolic activity and walking speed, even though boundary conditions differed from reference conditions.^7^ Bröde et al. (2016) adapted the UTCI scale based on different metabolic activity levels and duration of exposure. This paper focused on trips walked in the urban environment, that can reasonably be assumed shorter than one hour; for such limited exposure times, the difference between results of the cited work and our parameters was small, therefore we did not adopt adjusted UTCI values as heat stress threshold. Nevertheless, for specific cases, such as people performing intense physical activity or continuous exposure to DSR for several hours, adapting the heat stress threshold would be recommended.

The presented methodology would be valid to define minimum requirements of DSR exposure in winter, to compensate for cold stress. By coupling summer and winter scenarios, design solutions to make pedestrian paths responsive to seasonal changes could thus be proposed. During the cold season, DSR is much reduced compared to summertime; at the same time, a pleasant warm sensation can have a large impact on thermal comfort even if solar energy is low, while also being crucial for gaining vitamin D. Being based on physiology, this research does not take into consideration the psychological component of thermal comfort, or behavioural adaptation (Nikolopoulou and Steemers 2003). Since urban designers are the target of the proposed workflow, a different perspective is presented: the goal is to maximise potential comfort outdoors for a variety of users.

## Conclusions

This paper has provided a framework to assess the impact of DSR on pedestrians, by proposing a simplified way to evaluate DSR exposure based on environmental conditions and metabolic activity of users of diverse walking abilities. It has compared the energy intake that a walking person could absorb without feeling exhausted to the equivalent amount of DSR that a pedestrian is exposed to. In this way, the maximum energy capacity concept has been used to identify the maximum value of solar radiation energy intake of the human body before reaching the exhaustion threshold. The workflow has been specifically developed for urban designers and planners in their professional practice, to encourage more inclusivity and focus on users in cities.

Two user profiles have been delineated, a young adult and an elderly person with mobility impairment, characterised by metabolic activity, walking speed and maximum energy capacity. The results highlighted that younger adults have an energy budget to cope with DSR three times higher than elderly people. This threshold was compared to thermal stress simulated during clear-sky summer hours to assess walkability, defined as microclimatic conditions allowing pedestrians to walk without feeling exhausted. The framework has been tested in two different environments in Milan, an open area and an urban canyon with a H/W ratio of 1.0 to demonstrate its applicability.

Limitations of the proposed methodology were mainly linked to the tools used for microclimatic calculations. The accuracy of results could be improved using more sophisticated software and workflows: for example, modelling surface materials through Ladybug tools would improve T_mrt_ calculations, yet the contribution of R^*^ would keep the DSR exposure graph relevant. The user profile catalogue could be expanded to account for diverse user groups and mobility requirements, which would benefit from collaboration across disciplines. Finally, the proposed methodology could be applied to evaluate the thermal stress of pedestrian paths in existing settings and design proposals in cities of diverse climate zone, adapting relevant thresholds.

## Figures and Tables

**Fig. 10:**
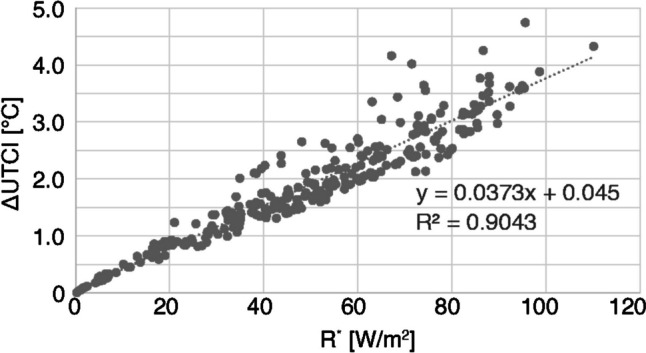
Linear regression analysis between R^*^ and ΔUTCI. Data refer to the open area exercise

**Fig. 11:**
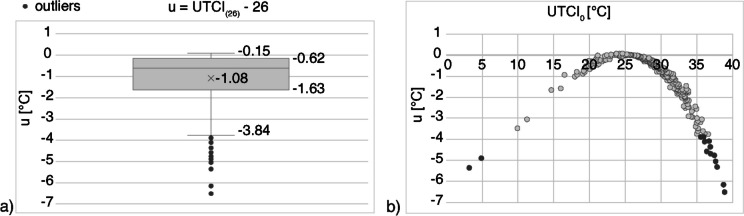
(a) Box plot presenting the distribution of the variable u, defined as the difference between UTCI_(26)_ (calculated via R0) and 26 °C; (b) relation between UTCI_0_ and u, showing that outliers are hours in which UTCI value in shade (UTCI_0_) is far from the threshold value (in this case, 26 °C)

**Fig. 12:**
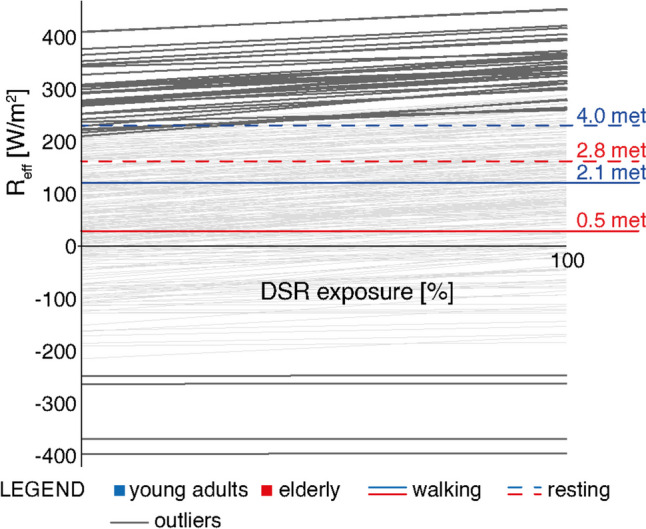
Position of hours labelled as outliers in the DSR exposure graph. Since they are located at large distance from the x axis (R_eff_ = 0), it is reasonable to conclude that this result does not affect the outcome of the DSR exposure graph categorisation

## Appendix A

### Statistical analysis to test the validity of the proposed model

Equation (6) is formulated assuming a linear relationship between R^*^ and ΔUTCI. Figure 10 presents a linear regression analysis between the two variables based on the open area exercise.

To evaluate the accuracy in estimating the variable R_0_ via Eq. 6, an inverse process was applied: in Eq. 4, R^*^ was substituted with R_0_, and T^*^_mrt_ with T_mrt_. A new T_mrt_ value was calculated (T_mrt(26)_) as the mean radiant temperature that, considering constant the other environmental parameters and simulating a shaded condition, would result in a UTCI value of 26 °C:

9 $${T}_{mrt(26)}={\left[{T}_{mrt}^{4}-\frac{{R}_{0}}{\left({\varepsilon }_{p}\cdot \sigma \right)}\right]}^{0.25}$$

Then, T_mrt(26)_ was used to calculate UTCI_(26)_: based on the proposed model, UTCI_(26)_ should be equal to 26 °C. The new variable ‘u’ was defined as the difference between UTCI_(26)_ and 26 °C; Figure 11a presents statistical analysis of u, illustrating the distribution of results. A total of 39 outliers were found (14%). Therefore, further investigation about whether this result would affect the outcome of the DSR exposure graph was performed. Figure 11b presents the relation between u and UTCI_0_: all outliers corresponded to hours in which microclimatic conditions in shade were largely distant from the heat stress threshold (26 °C). Thus, since all hours labelled as outliers were positioned at the extremes of the DSR exposure graph (Fig. 12), it was possible to conclude that outliers would not affect the categorisation of microclimatic conditions based on walkability.

Based on this evaluation, the proposed model is a valid tool to evaluate the impact of DSR exposure on pedestrians when microclimatic conditions are not largely different from the selected thermal stress threshold.

## Appendix B

### Calculation process for two case study hours

The calculation process about the two case study hours isolated in Fig. 8 is presented. The first set of calculations refers to hour ‘m’,and the second set refers to hour ‘n’.

#### Hour ‘m’: 6^th^ July, 10 am DST

Input data from EnergyPlus file:

**Table:**

Dry bulb temperature [°C]	Relative humidity [%]	Wind speed [m/s]	Total sky cover (tenths)
20.9	59	1	0

**Table:**

Direct normal radiation (I) [W/m^2^]	Diffuse horizontal radiation [W/m^2^]	Horizontal infrared radiation intensity [W/m^2^]
526	161	348

LB *Sunpath*: altitude = 42.4﻿°

10 $${\mathrm{f}}_{\mathrm{p}}=0.233$$

11 $${\mathrm{R}}^{*}=\mathrm{I}\cdot {\mathrm{a}}_{\mathrm{k}}\cdot {\mathrm{f}}_{\mathrm{p}}=526\cdot 0.7\cdot 0.233 = 85.88\mathrm{ W}/\mathrm{m}2$$

LB *OutdoorSolarMRT*: T_mrt_ = 33.4 °C.

LB *UTCI*: UTCI_0_ = 24.6 °C

12 $${\mathrm{T}}_{\mathrm{mrt}}^{*}={\left[{\mathrm{T}}_{\mathrm{mrt}}^{4}+\frac{{\mathrm{R}}^{*}}{\left({\upvarepsilon }_{\mathrm{p}}\cdot\upsigma \right)}\right]}^{0.25}= {\left[{(33.4+273.15)}^{4}+\frac{85.88}{\left(0.95\cdot\upsigma \right)}\right]}^{0.25}-273.15=46.4 ^\circ \mathrm{C}$$

LB *UTCI*: UTCI_1_ = 28.4 °C.

ΔUTCI = 28.4—24.6 = 3.8 °C.

13 $${\mathrm{R}}_{0}=\frac{{(\mathrm{UTCI}}_{0}-26) \cdot {\mathrm{R}}^{*}}{\Delta \mathrm{UTCI}}= \frac{(24.6-26) \cdot 85.88}{3.8} = -31.6\mathrm{ W}/\mathrm{m}2$$

14 $${\mathrm{R}}_{\mathrm{eff}}={{\mathrm{R}}^{*}+\mathrm{R}}_{0} = 85.88-31.6 = 54.3\mathrm{ W}/\mathrm{m}2$$

15 $${\mathrm{R}}_{\mathrm{eff}}= \frac{{\mathrm{R}}^{*}}{100}\mathrm{expo}+{\mathrm{R}}_{0}=\frac{85.88}{100}\mathrm{expo}-31.6$$

Key points on DSR exposure graph:

- Intersection with x-axis (heat stress threshold): ${\mathrm{R}}_{\mathrm{eff}}=0\mathrm{ W}/{\mathrm{m}}^{2}= \frac{85.88}{100}\mathrm{expo}-31.6$ > > expo = 37%
- Walking adaptation threshold for the elderly: ${\mathrm{R}}_{\mathrm{eff}}=29\mathrm{ W}/{\mathrm{m}}^{2}= \frac{85.88}{100}\mathrm{expo}-31.6$ > > expo = 71%
- Walking adaptation threshold for young adults: ${\mathrm{R}}_{\mathrm{eff}}=122\mathrm{ W}/{\mathrm{m}}^{2}= \frac{85.88}{100}\mathrm{expo}-31.6$ > > expo = 179%

#### Hour ‘n’: 27^th^ August, 12 pm DST

Input data from EnergyPlus file:

**Table:**

Dry bulb temperature [°C]	Relative humidity [%]	Wind speed [m/s]	Total sky cover (tenths)
23.1	62	0	0

**Table:**

Direct normal radiation (I) [W/m^2^]	Diffuse horizontal radiation [W/m^2^]	Horizontal infrared radiation intensity [W/m^2^]
511	232	362

LB *Sunpath*: altitude = 50.2°

16 $${\mathrm{f}}_{\mathrm{p}}=0.208$$

17 $${\mathrm{R}}^{*}=\mathrm{I}\cdot {\mathrm{a}}_{\mathrm{k}}\cdot {\mathrm{f}}_{\mathrm{p}}=511\cdot 0.7\cdot 0.208 = 74.38\mathrm{ W}/{\mathrm{m}}^{2}$$

LB *OutdoorSolarMRT*: T_mrt_ = 41.9 °C.

LB *UTCI*: UTCI_0_ = 29.1 °C

18 $${\mathrm{T}}_{\mathrm{mrt}}^{*}={\left[{\mathrm{T}}_{\mathrm{mrt}}^{4}+\frac{{\mathrm{R}}^{*}}{\left({\upvarepsilon }_{\mathrm{p}}\cdot\upsigma \right)}\right]}^{0.25}= {\left[{(41.9+273.15)}^{4}+\frac{74.38}{\left(0.95\cdot\upsigma \right)}\right]}^{0.25}-273.15=52.4 ^\circ \mathrm{C}$$

LB *UTCI*: UTCI_1_ = 31.9 °C.

ΔUTCI = 31.9 – 29.1 = 2.8 °C.

19 $${\mathrm{R}}_{0}=\frac{{(\mathrm{UTCI}}_{0}-26) \cdot {\mathrm{R}}^{*}}{\Delta \mathrm{UTCI}}= \frac{(29.1-26) \cdot 74.38}{2.8} = 82.3\mathrm{ W}/{\mathrm{m}}^{2}$$

20 $${\mathrm{R}}_{\mathrm{eff}}={{\mathrm{R}}^{*}+\mathrm{R}}_{0} = 74.38+82.3 = 156.7\mathrm{ W}/{\mathrm{m}}^{2}$$

21 $${\mathrm{R}}_{\mathrm{eff}}= \frac{{\mathrm{R}}^{*}}{100}\mathrm{expo}+{\mathrm{R}}_{0}=\frac{74.38}{100}\mathrm{expo}+82.3$$

Key points on DSR exposure graph:

- Intersection with x-axis (heat stress threshold): ${\mathrm{R}}_{\mathrm{eff}}=0\frac{\mathrm{W}}{{\mathrm{m}}^{2}}= \frac{74.38}{100}\mathrm{expo}+82.3$ > > expo = -110%
- Walking adaptation threshold for the elderly: ${\mathrm{R}}_{\mathrm{eff}}=29\frac{\mathrm{W}}{{\mathrm{m}}^{2}}= \frac{74.38}{100}\mathrm{expo}+82.3$ > > expo = -70%
- Walking adaptation threshold for young adults: ${\mathrm{R}}_{\mathrm{eff}}=122\frac{\mathrm{W}}{{\mathrm{m}}^{2}}= \frac{74.38}{100}\mathrm{expo}+82.3$ > > expo = 55%

## Abbreviations

DSR: Direct Solar Radiation
DST: Daylight Saving Time
T_mrt_: Mean Radiant Temperature
UTCI: Universal Thermal Climate Index

## Footnotes

1 It should be noted that f_p_ decreases as γ increases: in fact, when the sun is high in the sky, the surface of a person exposed to DSR is reduced.

2 Mean radiant temperature calculated including DSR as an additional source of energy.

3 Mean radiant temperature calculated as the sum of the fluxes received from surrounding surfaces (i.e., for a given surface i, emitted thermal radiation, E_i_, and diffusely reflected solar radiation, D_i_) plus the diffuse solar radiation from the sky.

4 Conversion process: 0.225 ml O_2_/kg/m * 0.79 m/s = 0.17775 ml O_2_/kg/s * 60 s = 10.665 ml O_2_/kg/minute / 3.5 ml O_2_/kg/minute = 3.0 met.

5 These values correspond to the difference between the exhaustion threshold (Fig. 3a) and the energy cost of resting sitting (Table 1).

6 It should be noted that.epw files report DSR as energy (measured in Wh/m^2^), not power. Since radiation is reported as cumulative over an hour, the provided values can be reasonable considered as irradiance (W/m^2^): as such, this conversion will be used in the following calculations. As per Ladybug components formulation, during computation processes, variables in a specific hour are associated with solar radiation collected during the 60 min after the selected time.

7 Reference conditions: walking speed of 1.1 m/s, metabolic activity of 2.3 met (135 W/m^2^), exposure time of 2 h.
